# Supplementary material for: A regional One Health approach to the risk of invasion by Anopheles stephensi in Mauritius
Source: PLoS Negl Trop Dis. 2024 Sep 11;18(9):e0011827. doi: 10.1371/journal.pntd.0011827 (PMC11444417; doi:10.1371/journal.pntd.0011827)
Supplement: S1 Text — Included methods used to determine the number of spot checks used per month per district. (DOCX) [file pntd.0011827.s001.docx]

**Supporting Information**

***Larval survey methods***

Since human population density is a major factor influencing vector-borne disease risk transmission in Mauritius, this parameter was used to estimate the relative frequency at which spot check surveys need to be carried out in the districts by the Health Authority. Taking into consideration the vast extent of uninhabited (and hence epidemiologically irrelevant) lands in Mauritius, population density within inhabited areas were considered to evaluate the risk of disease transmission. To obtain this parameter, population estimate by district (as per the national Health Statistics report) was divided by the surface area of inhabited land in the district (which was calculated by creating a vector layer of inhabited lands on QGIS 3.16). The district with the smallest population density was assigned a risk factor of 1 and proportionately extrapolated to the other districts (see Table 1 below). Based on the Disease Risk Index, the number of monthly spot check surveys recommended by district was calculated using the following formula:

Table 3.2: Demographic information and recommended number of *Ae. albopictus* spot check surveys by district in one month during inter-epidemic situation. (Data Source: Health Statistics report, 2019)

| **District** | **Inhabited Area (Km^2^)** | **Population size (Persons)** | **Population density (Persons/Km^2^ of inhabited area)** | **Disease Risk Index** | **Recommended no. of spot check surveys** |
| --- | --- | --- | --- | --- | --- |
| Port Louis | 18.7 | 118455 | 6332 | 3 | 6 |
| Plaines Wilhems | 73.5 | 366961 | 4994 | 2 | 5 |
| Moka | 30.7 | 138736 | 4520 | 2 | 5 |
| Flacq | 37.7 | 138736 | 3679 | 2 | 4 |
| Grand Port | 31.6 | 112847 | 3575 | 2 | 4 |
| Savanne | 21.2 | 68309 | 3216 | 1 | 3 |
| Pamplemousses | 48.8 | 141586 | 2902 | 1 | 3 |
| Riviere du Rempart | 41.9 | 108009 | 2578 | 1 | 3 |
| Black River | 38.2 | 83765 | 2194 | 1 | 2 |

Larval surveys were carried out by Health Surveillance Officers of the Vector Biology and Control Division - a national department under the Ministry of Health and Wellness. By virtue of their profession, each Health Surveillance Officer has a ‘access pass’ issued by the State that they show to residents to facilitate their entry in private, para-static and public premises. Yards are surveyed only after authorization have been verbally obtained by the owners. Mosquito surveys are conducted by the Health Surveillance Officers in the presence/assistance of the owners and relevant information are obtained from the latter. Larval surveys were conducted on both private, para-static and public properties.

These observational data were collected by trained Health Surveillance Officers of the Ministry of Health when conducting the larval surveys. Sites positive for mosquito larvae were described on field survey forms and classified accordingly.

**References**

1. (MOH) MoHaW. Mauritius health statistics record 2019. 2019
